# Protocol for achieving enhanced snRNA-seq data quality using Quality Clustering

**DOI:** 10.1016/j.xpro.2025.103717

**Published:** 2025-03-29

**Authors:** Paavo J. Tavi, Johannes Ojanen, Pia Laitinen, Suvi Linna-Kuosmanen

**Affiliations:** 1 A. I. Virtanen Institute for Molecular Sciences, University of Eastern Finland, 70211 Kuopio, Finland

**Keywords:** Bioinformatics, Single Cell, RNA-seq, Computer sciences

## Abstract

Single-nucleus RNA sequencing (snRNA-seq) data analysis presents a challenge in samples that have high levels of ambient RNA contamination. Quality Clustering (QClus) removes empty and highly contaminated droplets by utilizing multiple contamination metrics. Here, we present the steps for snRNA-seq data preprocessing using the QClus algorithm. First, we describe how to set up a computational environment. Next, we demonstrate how to use QClus to remove highly contaminated droplets, and finally, we show how to visualize and evaluate the results.

For complete details on the use and execution of this protocol, please refer to Schmauch et al.^1^

## Highlights

• Instructions for QClus setup for preprocessing of snRNA-seq samples
• Application of unspliced RNA counts for improved data quality
• Steps to run QClus to filter out contamination
• Guidance on QC step visualization for protocol evaluation

## Abstract (editor highlights)

Publisher’s note: Undertaking any experimental protocol requires adherence to local institutional guidelines for laboratory safety and ethics.

## Graphical abstract

**Figure:**
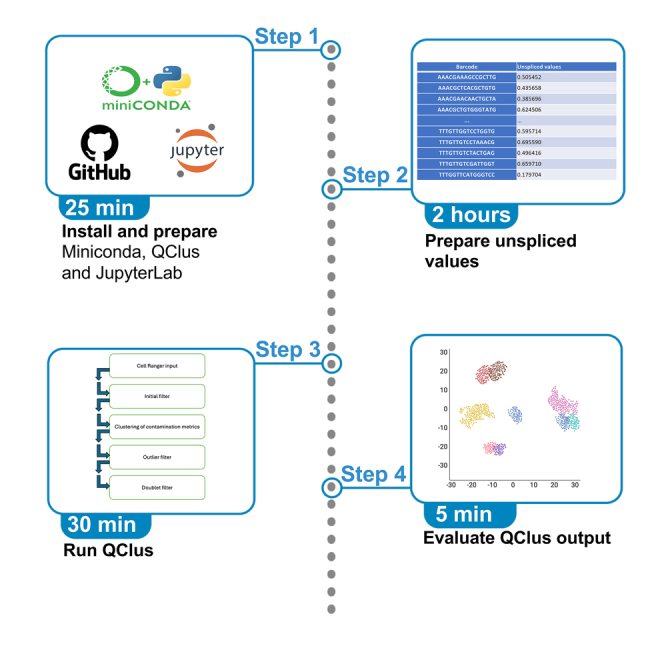

## Before you begin

In certain tissues, droplet-based snRNA-seq analysis remains challenging due to high levels of ambient RNA contamination. This ambient RNA can stem from, for example, cytoplasmic or cell-free RNA. In cardiac tissues, high levels of contamination originate from cardiomyocytes (CMs). These contractile units of the heart are large and contain high numbers of mitochondria. Due to their size, metabolic activity, and mitochondrial count, CMs produce a high amount of cytosolic RNA and cell debris when nuclei are isolated. Contamination originating from CMs can be mistaken as a genuine signal from other cell types, leading to a misinterpretation of expression patterns. Therefore, removing empty as well as highly contaminated droplets during preprocessing is crucial for accurate expression analysis. To meet these challenges in heart and other difficult tissues, snRNA-seq analysis workflows require rigorous quality control.

In this protocol, we outline the steps involved in snRNA-seq data quality control using the QClus droplet filtering algorithm. QClus produces high-quality data from snRNA-seq samples for downstream analysis while minimizing user input through automation.

For complete details and use, please refer to Schmauch et al.^1^ and the project GitHub page (https://github.com/linnalab/qclus).

### Hardware preparation

The QClus algorithm is implemented as a python package and can be run on Linux, MacOS or Windows. Certain steps might take longer than estimated depending on computational resources and sample size.

### Software preparation

To build a computational Python environment this protocol uses Miniconda v.24.4.0. and an integrated development environment JupyterLab v.4.2.3. The system requirements for Miniconda v.24.4.0 can be found from https://docs.anaconda.com/miniconda/miniconda-system-requirements/. Other integrated development environments (IDEs) can be used as well. For the Miniconda and your chosen IDE we recommend using the newest version that is compatible with your device.

### Setting up the computational environment

**Timing: 10–25 min**

1. Install Miniconda.
  a. Download Miniconda to your chosen device from https://docs.anaconda.com/miniconda/.

***Note:*** You can also use Anaconda, but it contains default packages that are not necessary for the analysis.

2. Set up a virtual environment and install QClus.
  a. Open the command line in your chosen device.
  b. Go to https://github.com/linnalab/qclus and copy the first command under installation to your command line and press enter.
    >git clone https://github.com/linnalab/qclus.git
  c. Change directory to qclus.
    >cd qclus
  d. Copy the second command under installation to your command line and press enter to create an environment called qclus and download packages.
    >bash environment.sh
  e. Activate qclus environment.
    >conda activate qclus
    ***Note:*** In the future QClus will be added to PyPI, making it available via pip install. For the latest instructions refer to the project GitHub page (https://github.com/linnalab/qclus).

3. Launch JupyterLab. This opens JupyterLab in your browser.

>jupyter lab

***Note:*** JupyterLab is installed when you create the qclus environment. Instead of JupyterLab, you may also use other integrated development environments (IDEs) such as VScode or PyCharm.

***Note:*** As you launch JupyterLab in the qclus directory, the QClus information pages and tutorials are visible in JupyterLab. You should utilize them as needed during the workflow.

## Key resources table

**Table:** 

REAGENT or RESOURCE	SOURCE	IDENTIFIER
**Software and algorithms**		

Miniconda v.24.4.0	Python Software Foundation	https://docs.anaconda.com/miniconda/
JupyterLab v.4.2.3	Project Jupyter	https://pypi.org/project/jupyterlab/
QClus v.0.1	Schmauch et al.^1^	https://github.com/linnalab/qclus
scanpy v.1.10	Wolf et al.^2^	https://pypi.org/project/scanpy/
pandas v.2.2.2	McKinney^3^	https://github.com/pandas-dev/pandas/tree/v2.2.2
Matplotlib v.3.9.1	Hunter^4^	https://pypi.org/project/matplotlib/

## Step-by-step method details

In this section, we describe the major steps involved in the use of QClus.

***Note:*** All the code presented in this section is available in the tutorial files that you can access in the JupyterLab-window or go to https://github.com/linnalab/qclus/tree/main/tutorials. You can copy the code from the tutorial file to run your analysis.

### Preparing unspliced values

**Timing: 15 min–2 h**

In this section we prepare the unspliced values from the snRNA-seq data. You need unspliced values to run QClus.

***Note:*** These steps are not required if you already have your unspliced values. If this is the case, go directly to step 3. You only need step 1 or 2 to obtain your unspliced values depending on whether you have run Velocyto^5^ on your data.

1. Get unspliced values from the .loom file after running Velocyto^5^ on your data.
  a. Go to tutorials folder and “splicing _from_loompy.ipynb”notebook in your JupyterLab window or go to https://github.com/linnalab/qclus/blob/main/tutorials/splicing_from_loompy.ipynb.
  b. Run the code in the tutorial file or copy the code to a new Jupyter notebook file and run the code to obtain unspliced values.
2. Get unspliced values from the sorted BAM file produced by Cell Ranger^6^ for your data.
  a. Go to tutorials folder and “splicing_from_bam.ipynb”notebook in your Jupyter lab window or go to https://github.com/linnalab/qclus/blob/main/tutorials/splicing_from_bam.ipynb.
  b. Run the code in the tutorial file or copy the code to a new Jupyter notebook file and run the code to obtain unspliced values.

***Note:*** When writing the path to bam or loom file, modify the path structure shown in the tutorial according to your file location.

### Running QClus on snRNA-seq data

**Timing: 10–30 min**

In this step, we will run the QClus on the snRNA-seq data using the default parameter values used in Schmauch et al.^1^ Marker genes and metrics used in the preprocessing are specific to cardiac tissue.

3. Open a new Jupyter Notebook file from the launcher by clicking the icon under “Notebook” or from File-> New->Notebook.
  a. Name the file appropriately.
4. Import packages using the import command as shown in the “qclus_tutorial.ipynb” file and below.

>%load_ext autoreload

>%autoreload 2

>import sys

>sys.path.append(’..’)

>import qclus as qc

>from qclus.gene_lists import ∗

>from qclus.utils import ∗

>import scanpy as sc

>import pandas as pd

>import matplotlib.pyplot as plt

>import warnings

>warnings.filterwarnings(“ignore”)

5. Assign snRNA-seq data and unspliced fraction data to variables counts_path and splicing_path.

***Note:*** When assigning data files to variables, modify the path structure according to your file location and directory structure.

>splicing_path = ”../../fraction_unspliced_loom.csv”

>counts_path = “../../samples/sample_lq.h5”

***Note:*** QClus v.0.1 expects the counts_path variable to point to the filtered .h5 file. Other inputs will be supported in later implementations.

6. Read the fraction_unspliced file from csv format to a data frame and assign this data frame to a variable fraction_unspliced.

>fraction_unspliced = pd.read_csv(splicing_path, index_col=0)

***Note:*** You can check the fraction_unspliced data frame by calling the fraction_unspliced variable.

7. Assign the output of the QClus to an AnnData object called adata and run the qc.run_qclus function as shown in the “qclus_tutorial.ipynb”-file.

>adata = qc.run_qclus(counts_path, fraction_unspliced)

***Note:*** The default parameter values used are conservative estimations and should yield high quality data for most snRNA-seq samples from cardiac tissues.^1^ However, they can be adjusted depending on the dataset.

***Note:*** The cardiac tissue specific and nuclei specific markers used in the analysis can be found from “gene_list.py”-file in the qclus folder in your JupyterLab window.

***Note:*** You can check the AnnData object by calling adata.

### Filtering and visualization of the QClus output

**Timing: 5 min**

In this step, we will check and evaluate the QClus output data using scanpy,^2^ pandas,^3^ and matplotlib.^4^

***Note:*** These steps are just one way of visualizing, filtering and evaluating the QClus output. You may prefer other methods for your data and workflow.

8. Check the qclus output by using pandas value.counts function (Table 1).

**Table 1:** QClus output using pandas value_counts function

Qclus output	
Initial filter	4236
Clustering filter	4231
outlier filter	5769
scrublet filter	2615
passed	8245

**Figure 1:**
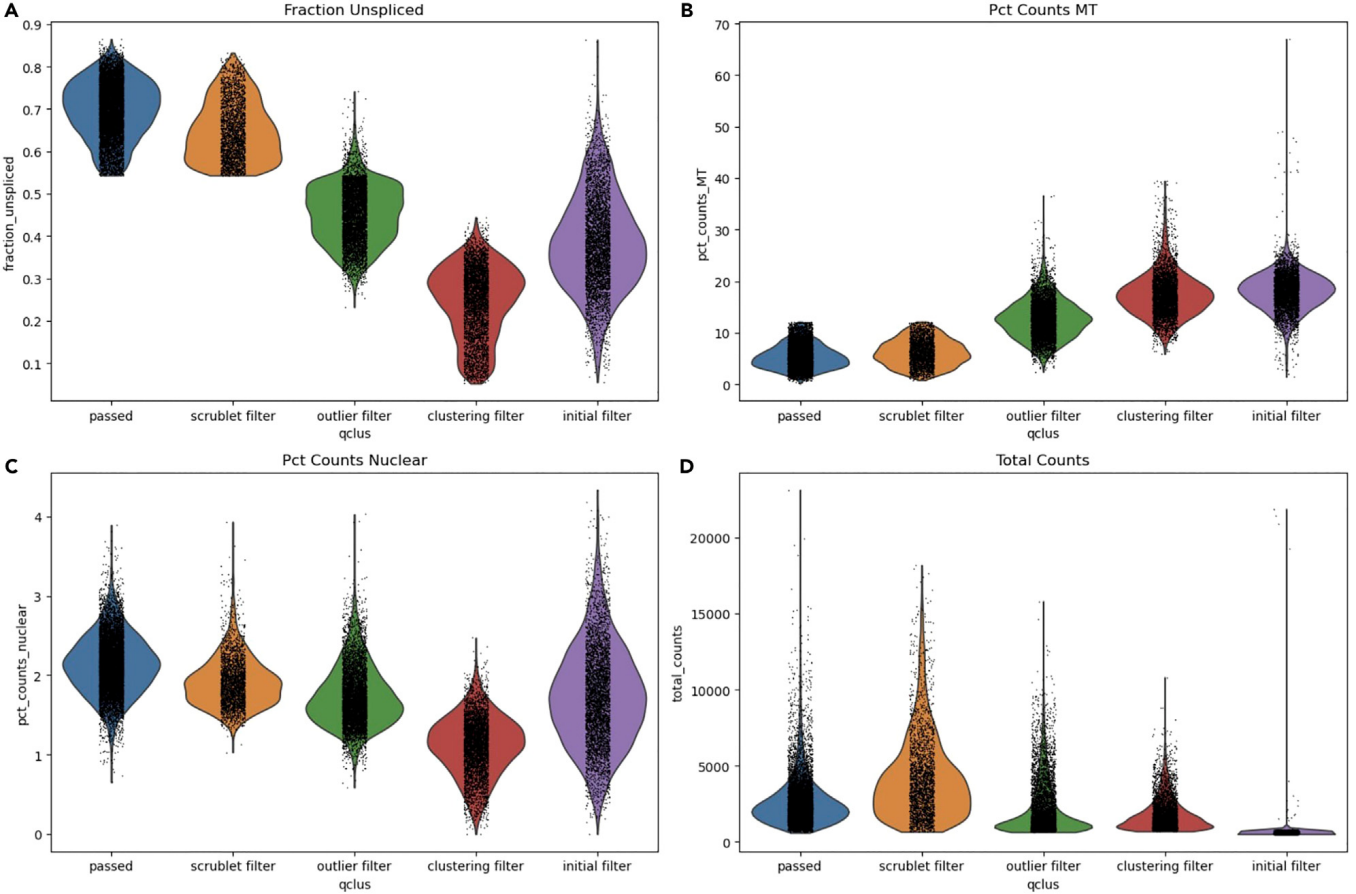
Violin plots representing each step of the QClus pipeline using four different keys (A) Violin plot of the filtering steps with fraction_unspliced. (B) Violin plot of the filtering steps with pct_counts_MT. (C) Violin plot of the filtering steps with pct_counts_nuclear. (D) Violin plot of the filtering steps with total_counts.

>adata.obs.qclus.value_counts()

***Optional:*** You can plot the filtering steps as violin subplots. As keys you can use for example fraction_unspliced, pct_counts_MT, pct_counts_nuclear and total_counts to see how these are filtered in each step of the pipeline (Figure 1).

>desired_order = ['passed', 'scrublet filter', 'outlier filter', 'clustering filter', 'initial filter']

>unique_qclus_values = adata.obs['qclus'].unique().tolist()

>qclus_order = [category for category in desired_order if category in unique_qclus_values]

>adata.obs['qclus'] = adata.obs['qclus'].astype('category')

>adata.obs['qclus'] = adata.obs['qclus'].cat.reorder_categories(qclus_order, ordered=True)

>fig, axs = plt.subplots(2, 2, figsize=(15, 10))

>sc.pl.violin(adata, 'fraction_unspliced', groupby='qclus', order=qclus_order, ax=axs[0, 0], show=False)

>axs[0, 0].set_title('Fraction Unspliced')

>sc.pl.violin(adata, 'pct_counts_MT', groupby='qclus', order=qclus_order, ax=axs[0, 1], show=False)

>axs[0, 1].set_title('Pct Counts MT')

>sc.pl.violin(adata, 'pct_counts_nuclear', groupby='qclus', order=qclus_order, ax=axs[1, 0], show=False)

>axs[1, 0].set_title('Pct Counts Nuclear')

>sc.pl.violin(adata, 'total_counts', groupby='qclus', order=qclus_order, ax=axs[1, 1], show=False)

>axs[1, 1].set_title('Total Counts')

>plt.tight_layout()

>plt.show()

9. Filter out initial_filter annotated cells from the AnnData object.

>adata = adata[adata.obs.qclus!=”initial filter”]

>adata.obsm[“Qclus_umap”] = adata.uns[“Qclus_umap”]

**Figure 2:**
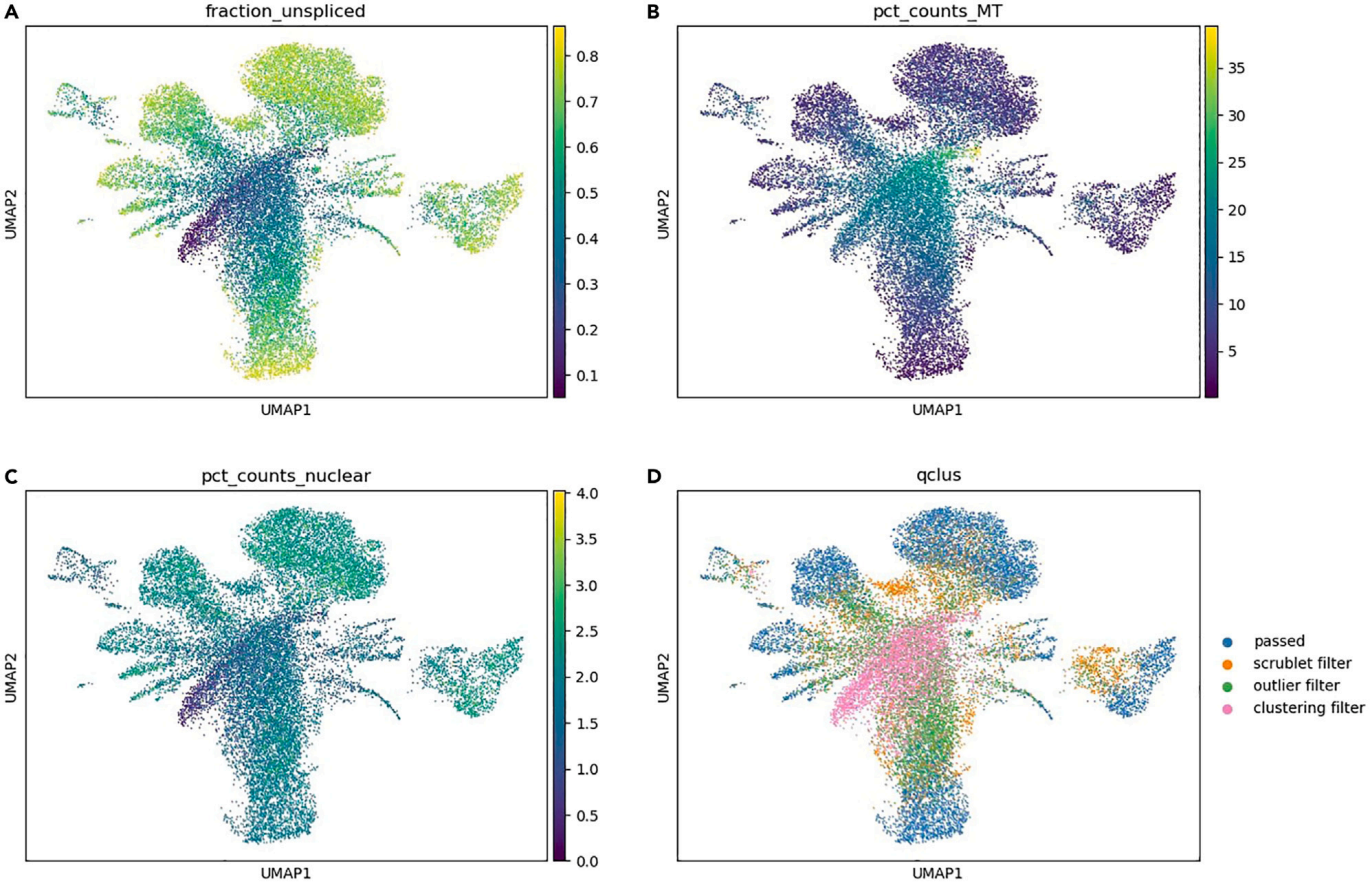
Unfiltered transcriptomic UMAP using four different keys (A) Unfiltered transcriptomic UMAP with fraction_unspliced. (B) Unfiltered transcriptomic UMAP with pct_counts_MT. (C) Unfiltered transcriptomic UMAP with pct_counts_nuclear. (D) Unfiltered transcriptomic UMAP with QClus.

10. Run standard processing for visualization using scanpy,^2^ save the adata.raw dataset, filter genes and plot the unfiltered transcriptomic UMAP (Figure 2).

>sc.pp.normalize_total(adata, target_sum=1e4)

>sc.pp.log1p(adata)

>adata.raw = adata

>sc.pp.highly_variable_genes(adata, min_mean=0.0125, max_mean=3, min_disp=0.5)

>sc.pp.filter_genes(adata, min_cells=10)

>adata = adata[:, adata.var.highly_variable]

>sc.pp.regress_out(adata, ['total_counts', 'pct_counts_MT'], n_jobs = 4)

>sc.pp.scale(adata, max_value=10)

>sc.tl.pca(adata, svd_solver='randomized')

>sc.pp.neighbors(adata, n_neighbors=10, n_pcs=40)

>sc.tl.umap(adata)

>sc.tl.leiden(adata, key_added="leiden")

>sc.pl.umap(adata, color=[“fraction_unspliced”, “pct_counts_MT”, “pct_counts_nuclear”, “qclus”], ncols=2)

**Figure 3:**
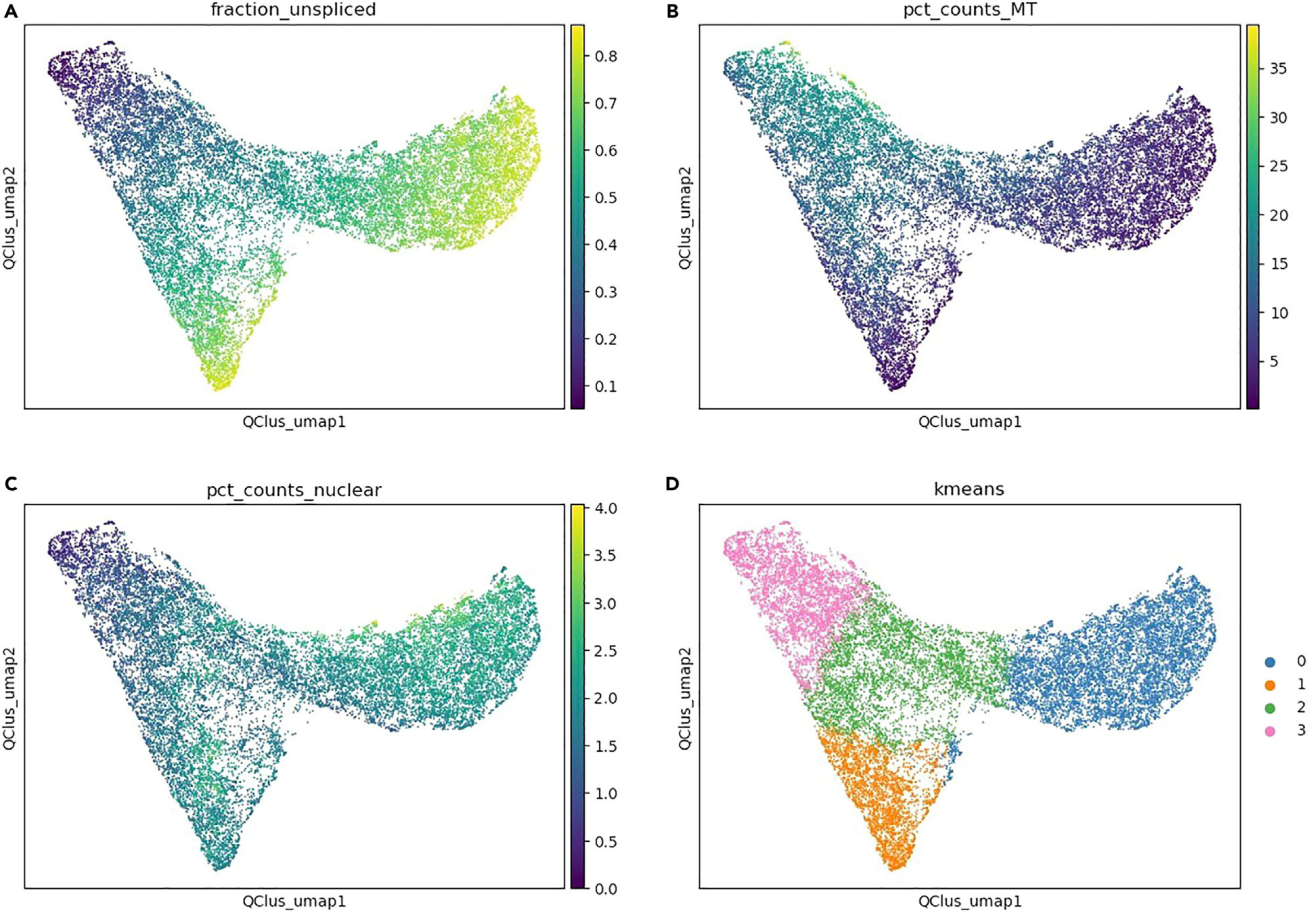
Scatter plot using QClus UMAP basis and four different keys (A) QClus UMAP with fraction_unspliced. (B) QClus UMAP with pct_counts_MT. (C) QClus UMAP with pct_counts_nuclear. (D) QClus UMAP with k-means.

11. Plot the QClus output as a scatter plot (Figure 3).

>%matplotlib inline

>sc.pl.embedding(adata, basis=”QClus_umap”, color=[“fraction_unspliced”, “pct_counts_MT”, “pct_counts_nuclear”, “kmeans”], ncols=2)

**Figure 4:**
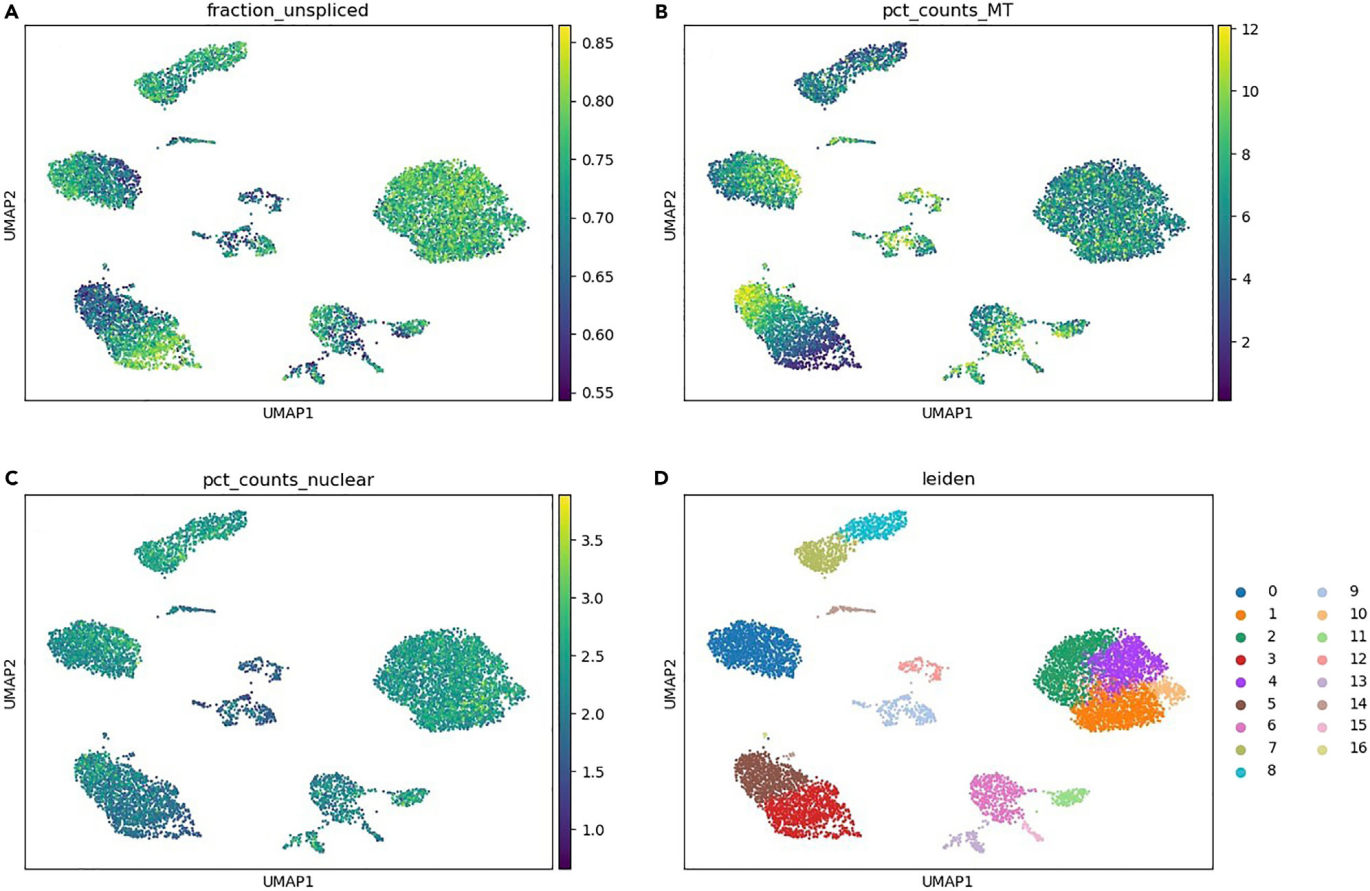
Filtered transcriptomic UMAP using passed values and four different keys (A) Filtered transcriptomic UMAP with fraction_unspliced. (B) Filtered transcriptomic UMAP with pct_counts_MT. (C) Filtered transcriptomic UMAP with pct_counts_nuclear. (D) Filtered transcriptomic UMAP with leiden.

12. Plot a filtered transcriptomic UMAP using passed values (Figure 4).

>adata = adata[adata.obs.qclus==”passed”]

>sc.tl.pca(adata, svd_solver='randomized')

>sc.pp.neighbors(adata, n_neighbors=10, n_pcs=40)

>sc.tl.umap(adata)

>sc.tl.leiden(adata, key_added=”leiden”)

>sc.pl.umap(adata, color=[“fraction_unspliced”, “pct_counts_MT”, “pct_counts_nuclear”, “leiden”], ncols=2)

***Note:*** The QClus output is saved to an AnnData object. This can be used for standard RNA-seq data downstream analysis.

## Expected outcomes

This protocol outlines the major steps involved in the use of the QClus droplet filtering algorithm. QClus filters out highly contaminated droplets and doublets from snRNA-seq data using several contamination metrics with very little manual input from the user. It also produces more high-quality samples from the same data when compared to other methods used for snRNA-seq data preprocessing.^1^ The specific number of samples filtered by each step of the QClus pipeline will depend on the quality of your data and the used parameter values. The default markers used here are cardiac tissue specific, but this method can be adapted for other tissues by changing the marker gene list or the input metrics. The QClus output, visualized in steps 8–12 is saved to an AnnData object that can be used for further downstream analysis such as clustering and cell type annotation.

## Limitations

At the time of writing, the QClus droplet-filtering algorithm works exclusively with 10× Genomics snRNA-seq data. As with any bioinformatic tool, QClus is limited in its ability to clean poor-quality data. Therefore, producing high quality snRNA-seq data for the analysis should remain a priority. This protocol uses QClus in a way that minimizes user input. However, the default parameter values might not be optimal for all datasets. If significant nuclei loss is observed during filtering or if separating cell types during downstream analysis is challenging, it may be necessary to adjust the parameter values to better suit your data. Optimizing parameter values can improve data quality in various other cases as well.

## Troubleshooting

### Problem 1

Loss of too many nuclei (related to steps 7 and 8).

### Potential solution

When you check the QClus output in step 8, you see how many nuclei the outlier filter removes based on the unspliced and mitochondrial fraction distribution (Table 1). If this amount is too high for your dataset, you might want to adjust the outlier filter parameters outlier_unspliced_diff and outlier_mito_diff to a higher value. This leads to less nuclei being considered as outliers based on unspliced and mitochondrial fractions distribution. You will see a decrease in the number of nuclei being filtered by the outlier filter in step 8 and a decrease in the size of the outlier filter cluster in the unfiltered transcriptomic UMAP (Figure 2D).

In addition, you can adjust the clusters_to_select parameter to include the lower quality cluster 3 as well. This will decrease the number of nuclei filtered by the clustering filter in step 8 (Table 1; Figure 1) and show a decrease in size of the clustering filter cluster in the unfiltered transcriptomic UMAP (Figure 2D). This will decrease the overall quality of the filtered sample.

You can also decrease the number of clusters produced by changing the parameter clustering_k to a lower value from the default 4. This should show a decrease in the number of nuclei being filtered by the clustering filter in step 8 (Table 1; Figure 1) and decrease the size of the clustering filter cluster in the unfiltered transcriptomic UMAP (Figure 2D), as well as remove cluster 3 from the QClus output scatter plot (Figure 3D).

All solutions presented above decrease the number of passed nuclei. To achieve the optimal balance between number of nuclei passed and the filtered data quality, you might have to adjust multiple metrics.

### Problem 2

Too many nuclei pass the filters (related to steps 7 and 8).

### Potential solution

If you notice that too many nuclei pass the filters and you suspect some of them are not of high quality, you can adjust the outlier filter parameters outlier_unspliced_diff and outlier_mito_diff to a lower value. This will lead to more nuclei being filtered by the outlier filter based on the distribution of unspliced and mitochondrial fractions in step 8 (Table 1; Figure 1). You can also see an increase in the size of the outlier filter cluster in the unfiltered transcriptomic UMAP (Figure 2D) and increase in the number of clusters produced in the filtered transcriptomic UMAP (Figure 4D).

In addition, you can adjust the clusters_to_select parameter to only include the best quality clusters 0 and 1 instead of the default 0, 1 and 2. This will significantly increase the number of nuclei filtered by the clustering filter in step 8 (Table 1; Figure 1), and show an increase in size of the clustering filter cluster in the unfiltered transcriptomic UMAP (Figure 2D), as well as produce more clusters in the filtered transcriptomic UMAP (Figure 4D). However, this might lead to the loss of some good quality nuclei.

You can also increase the number of clusters produced by changing the clustering_k parameter from the default value of 4. This should show an increase in the number of nuclei being filtered by the clustering filter in step 8 (Table 1; Figure 1) and it should increase the size of the clustering filter cluster in the unfiltered transcriptomic UMAP (Figure 2D) as well as add a cluster 4 to the QClus output scatter plot (Figure 3D). You should also see a higher number of clusters produced in the filtered transcriptomic UMAP (Figure 4D).

All solutions presented above will lead to fewer passed nuclei. You might have to adjust multiple parameters to get the optimal balance between the amounts of nuclei filtered and the quality of the passed nuclei.

### Problem 3

No developer tools found when running the git clone command (related to step 2 of before you begin).

### Potential solution

If you use a MacOS device to run this protocol, you need to install Xcode Command Line Tools to run the git clone command. If you run the command without Xcode, your device will suggest installing Xcode. Follow the install prompts to install Xcode and run the git clone command again after installation to proceed with the protocol. If the install prompt does not appear, you can do a manual install by running the following command in your command line.

>xcode-select --install

### Problem 4

Errors stemming from incorrect format of passed gene lists.

### Potential solution

A few things should be noted if you wish to pass custom gene lists to QClus. First, the nuclear gene list must be passed as a list, and the cell type gene list must be passed as a dictionary. The cell type name should be the key in the dictionary with each key corresponding to a list of gene names. Second, ensure you are passing gene names as strings that match the naming of genes in your data. This can sometimes be a problem if you are processing non-human data.

You do not need to match the default naming of the objects (“nucl_30” and “celltype_gene_set_dict”).

For an example of how to use QClus with custom gene lists, see our brain dataset processing tutorial at https://github.com/linnalab/qclus/tree/main/tutorials.

## Resource availability

### Lead contact

Further information and requests for resources and reagents should be directed to and will be fulfilled by the lead contact, Suvi Linna-Kuosmanen (suvi.linna-kuosmanen@uef.fi).

### Technical contact

Technical questions on executing this protocol should be directed to and will be answered by the technical contact, Johannes Ojanen (johanneo@uef.fi).

### Materials availability

As this work describes a computational protocol, it did not create any new materials.

### Data and code availability

The example sample that is processed in this protocol is taken from the CAREBANK dataset.^7^ The data are available via lead contact Suvi Linna-Kuosmanen (suvi.linna-kuosmanen@uef.fi). Code availability: GitHub: https://github.com/linnalab/qclus; Zenodo: https://doi.org/10.5281/zenodo.13773285.

## Acknowledgments

The computational resources were provided by the Bioinformatics Center of the University of Eastern Finland.

The project was funded by the Aarne Koskelo Foundation, the Finnish Foundation for Cardiovascular Research, the Academy of Finland (grant no. 342074), the Yrjö Jahnsson Foundation, and the Paavo Nurmi Foundation.

The example sample that is processed in this protocol is taken from the CAREBANK dataset.^7^ The data are available via the lead contact: suvi.linna-kuosmanen@uef.fi.

## Author contributions

P.J.T. (main author): text, figures, and data analysis. J.O.: supervision of technical execution of the QClus algorithm and revision of the manuscript. P.L.: revision. S.L.-K.: supervision, resources, and funding.

## Declaration of interests

The authors declare no competing interests.
